# Supplementary material for: Factors that influence safe water drinking practices among older persons in slums of Kampala: Analyzing disparities in boiling water
Source: PLoS One. 2023 Sep 22;18(9):e0291980. doi: 10.1371/journal.pone.0291980 (PMC10516437; doi:10.1371/journal.pone.0291980)
Supplement: Attachment — Submitted filename: Comments and questions.docx [file pone.0291980.s002.docx]

**Title: Safe water drinking practices among older persons in slums of Kampala: Analyzing gender disparities in boiling water, Manuscript Number: PONE-D-23-11738**

**This is an interesting title and the way of writing is appreciated.**

Still, there are comments and questions on this manuscript.

1. “The purpose of this study was to investigate the factors that influence boiling water practice …” so why not the topic include factors that influence…?
2. “The responses were coded as “yes” for those who reported that they boiled water…”, how do you categorize those respondents who boil water ‘rarely’, ‘sometimes’, ‘often’ etc. Yes/no, questions might not be appropriate to answer this research question.
3. This research question was good if supported by a bacteriological test to answer whether respondents really boil the water or not.
4. Your analysis was done using ‘multivariate’. Why do you prefer multivariate to multivariable some books/scholars stated that multivariate is for more than one outcome variable, but your response variable is one ‘boiling water (yes/no)’.
5. Why authors preferred the ‘Pearson Chi-squared test’ that binary logistic regression?
6. How does ‘quality of house’ act as a factor associated with ‘boil water’?
