# Supplementary material for: Factors that influence safe water drinking practices among older persons in slums of Kampala: Analyzing disparities in boiling water
Source: PLoS One. 2023 Sep 22;18(9):e0291980. doi: 10.1371/journal.pone.0291980 (PMC10516437; doi:10.1371/journal.pone.0291980)
Supplement: Attachment — Submitted filename: Reviewers.docx [file pone.0291980.s003.docx]

Reviewer #2: Line 244-246 there is a statement from this article link that needs to be paraphrased as it is word for word from the original source at <https://www.ncbi.nlm.nih.gov/pmc/articles/PMC7115273/> : "Relatedly, Allegranzi, Memish (50), argue that religious faith has made many important contributions to the ethics of health care and has helped focus the attention of health care providers on both the physical and spiritual nature of humans."

**Action: *We thank the reviewer for this important observation. We have paraphrased this.***

Minor grammatical and spelling errors remain present throughout the manuscript.

**Action: *We appreciate this comment. We have read through the manuscript and corrected accordingly.***

Line 363-365 discusses the predictors of boiling water, however the wording is unclear. I would recommend rephrasing it for consistency of your findings. "The other predictors of not boiling water to make it safe for drinking..." since older individuals living in a dilapidated house, unimproved water sources, and not receiving financial support were less likely to boil their drinking water.

**Action. *Thanks for this suggestion. This has been addressed as suggested***

Overall, the authors took time to implement the revisions suggested in the previous round which made this paper much stronger. All of my other suggestions and those by the previous reviewers have been adequately addressed.

**Action: *We appreciate this.***
